# Case Report: Diagnostic Value of Metagenomics Next Generation Sequencing in Intracranial Infection Caused by Mucor

**DOI:** 10.3389/fmed.2021.682758

**Published:** 2021-09-23

**Authors:** YuChen Liu, Jun Zhang, Bing Han, LiJuan Du, ZhaoYang Shi, ChunCheng Wang, Min Xu, YongGang Luo

**Affiliations:** ^1^Department of Gynecology, The First Affiliated Hospital of Zhengzhou University, Zhengzhou, China; ^2^Gene Hospital of Henan Province, Precision Medicine Center, The First Affiliated Hospital of Zhengzhou University, Zhengzhou, China; ^3^Department of Pharmacy, The First Affiliated Hospital of Zhengzhou University, Zhengzhou, China; ^4^Department of Intensive Care Unit, The First Affiliated Hospital of Zhengzhou University, Zhengzhou, China; ^5^Department of Clinical Laboratory, The First Affiliated Hospital of Zhengzhou University, Zhengzhou, China

**Keywords:** *mucormycosis*, *Lichtheimia ramosa*, metagenomics Next Generation Sequencing, precision treatment, intracranial infection

## Abstract

mNGS(metagenomics Next Generation Sequencing), as a novel culture-independent approach, demonstrated the capability of rapid, sensitive, and accurate pathogen identification. At present, there have been many case reports about the use of mNGS to assist in the diagnosis of bacterial, fungal, viral and parasitic infections and to guide clinicians to determine appropriate treatment. However, the clinical understanding of this technique is not comprehensive, and the experience of using it is relatively limited. We reported a 53-year-old man who was admitted to hospital with a high fever and headache. His inflammatory biomarkers were markedly elevated. Based on the clinical presentation, He was initially diagnosed as having an intracranial infection of unknown etiology and received empirical antibiotics and systemic supportive treatment. But these did not relieve his symptoms. Both the blood and CSF specimens were examined using traditional culture, serological testing, and mNGS. Traditional culture and serological testing produced negative results, while the mNGS revealed the presence of a potential pathogen, *mucor*, in the CSF specimen. Then targeted antifungal treatment was selected quickly and his temperature gradually returned to normal. Thus, we report the case in which mNGS was an auxiliary method to diagnose *mucormycosis*, and discuss this case in combination with relevant literature, in order to improve the clinical cognition of this technology.

## Introduction

*Mucormycosis*, also known as *zygomycosis*, is a highly invasive conditional fungal infection. It has low incidence, about 1.2 per 1 million people per year (1) and high mortality about 87% (2). Clinically, *mucormycosis* can be divided into many types, among which the rhino-orbito-cerebral (ROCM) is the most common type, accounting for about 75% of reported cases (3), that is, the fungus in the nasal cavity, nasal sinus mucosa accompanied by orbital and intracranial invasion (4).

The early symptoms of the disease are not typical, and it is difficult to be detected by traditional etiological detection methods, so the clinical diagnosis still faces great challenges and delayed treatment significantly affects patient prognosis (5). Therefore, it is crucial to rapidly and accurately identify the pathogens of *mucor*, and then optimize medical therapy. Clinically most patients with *mycosis* are treated empirically without a clear diagnosis. Given the risk of death due to delayed treatment, there is an urgent clinical need for a technology that can quickly and accurately identify pathogens.

Metagenomics Next Generation Sequencing (mNGS) is a new genomics-based pathogen detection technology, which has the advantages of high detection rate and fast detection speed. Thus, we report the case of intracranial infection caused by *mucor*, in which traditional culture was negative but mNGS provided a positive finding.

## Case Rescription

A 53-year-old man presented to our hospital with a 7-day history of fever and severe headache and a 1-day history of left extension of the tongue and slurred speech.11 days before arriving at our hospital, the patient developed periodontal pain and maxillofacial swelling without obvious inducement and received antibiotics in the local hospital. However, his symptoms were not significantly relieved, neither did his body temperature drop.

On admission, vital signs were shown as: body temperature was 37.2°C, respiration was 20 beats per minute, heart rate was 84 beats per minute, blood pressure was 158/81 mmHg. Physical examination revealed a 1 cm lesion near the gum of the patient's upper incisor; the right side of the light reflection disappeared and the left side is dull; the left nasolabial groove is shallow and the patient has a left extension of the tongue. Blood routine examination (BRE) showed that white blood cells were 27.62 × 109/L (neutrophil accounted for 89.1%). The procalcitonin (PCT) level was elevated to 0.120 ng/mL, C-reactive protein (CRP) reached 178.30 mg/L. Magnetic resonance imaging (MRI) showed a lesion in the brainstem. Hence, based on previous clinical experience, the patient was diagnosed preliminarily with brainstem encephalitis and purulent gingivitis. An initial treatment of meropenem (2g/8h iv) and linezolid (0.6g/12 h iv) had been empirically administrated. At the same time, enteral nutrition, reducing intracranial pressure, anti-heart failure, organ function protection, analgesia and other symptomatic treatment were given. But there was no significant relief of his symptoms.

In order to improve the etiological examination, both his peripheral blood and CSF specimens were examined to investigate possible etiology using traditional culture and mNGS. The CSF presented a colorless and transparent appearance, intracranial pressure was 600 mmH_2_0;cytology showed 46 × 106 leukocyte/L, glucose: 4.05mmol/L, chloride: 125.3 mmol/L, lactic acid: 2.90 mmol/L and there was no evidence of *fungal*, or *acid-fast bacilli* infection. Serological testing was performed, including 1,3-β-D glucan test (G test), galactomannan antigen test (GM test) and bacterial smear test; however they were all negative. Computerized tomography (CT) showed paranasal sinusitis and bilateral hypertrophic inferior turbinate. Consider the possibility of infection by a particular pathogen, empirical antifungal and antiviral therapy of ganciclovir intravenous (0.5g/12h iv) and voriconazole (400mg/12h iv) was given.

**Figure 1:**
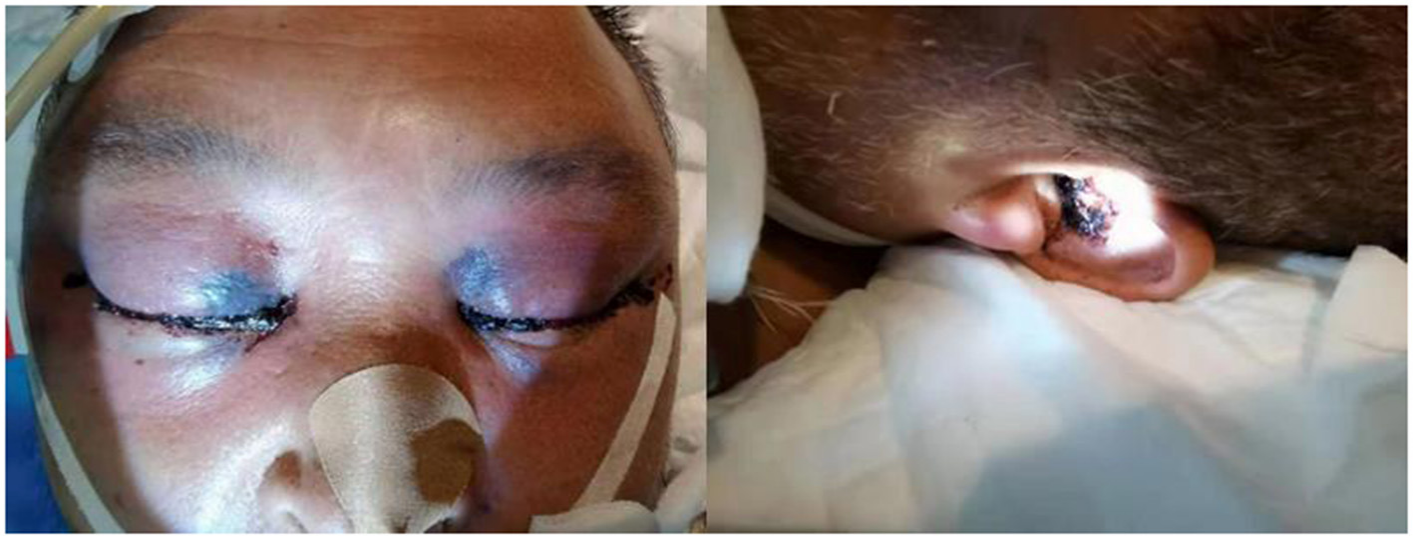
Black eschar lesions of the patient.

The fourth day after admission, the patient was unconscious and his temperature level reached 39.6°C. Black eschar lesions and bloody secretion are seen in the external auditory canal (Figure 1). Based on this, pathogen culture was performed on external auditory canal secretions. Laboratory tests to show CRP level to 340.50 mg/L. To exclude pulmonary infection, fiberoptic bronchoscopy was performed, and the results showed no significant abnormalities. MRI showed multiple intracranial soft tissue infections, sinus infections, soft tissue thickening of the posterior pharyngeal wall, suppurative otitis media, and gingival infection. Therefore, we detected nasal secretions by pathogen culture. Secretions of external auditory canal and nasal, peripheral blood and cerebrospinal fluid specimens were negative. However, within 48 h, mNGS revealed a total of 35 single-end reads in the genomic DNA of the CSF specimen, of which 4 reads were *Lichtheimia ramosa* (Table 1), and its relative abundance is 50%. Because the test results are consistent with the clinical manifestations, we adjusted the treatment regimen to amphotericin B liposomes intravenous (500 mg/24h iv). After a discussion with the otolaryngology department, we recommended surgical treatment for patient, but was rejected by the patient's family.

**Table 1:** The results of mNGS.

**Genus**			**Species**			
**Type**	**Name**	**Sequence number**	**Name**	**Sequence number**	**Realtive abundance**	**Attention**
G-	*Lichtheimia*	4	*Lichtheimia ramosa*	2	50.0%	High

On the eighth day of admission, after four days of conservative treatment, the patient's temperature was back to normal (37.2°C) but he suffered developed brainstem failure. We use high doses of vasoactive drugs and still have difficulty maintaining his blood pressure. After communicating with the patient's family, they requested to be discharged.

## Discussion

Here, we reported a case in which mNGS helped clinicians rapidly and accurately identifying *mucor* in a patient with intracranial infection. This patient was admitted to hospital due to high fever and headache. Traditional culture and serological testing did not determine possible infection etiology, while mNGS identified *mucor* in the CSF specimen. Combined with his clinical characteristics, his diagnosis was confirmed to be a ROCM.

### Route of Infection

This disease generally seen in people with malignant blood diseases, diabetes, organ transplantation and long term use of antibiotics, hormones, immunosuppressant. But a recent epidemiological study found that 19.16% of cases of *mucormycosis* occurred in patients with “no underlying disease” (6). This patient was a healthy adult before the onset of the disease and did not have any of the above risk factors. However, before the onset of the disease, he had received intravenous infusion treatment in a local primary hospital for “toothache and maxillofacial swelling”. Therefore, we suspect that the disease may be caused by the retrograde infection of pathogens along the bloodstream after tooth extraction.

### Clinical Manifestation

Early symptoms of ROCM are atypical and include runny nose, stuffy nose, headache, fever, facial swelling, vision loss, and mental distress. With the progression of the disease, persistent high fever, eye movement disorders, intracranial infection, and consciousness disorders may occur (7, 8). The typical presentation is sinusitis with painless black eschar formation in the face, nasal cavity or palate, but it is only seen in a few patients and suggests a poor prognosis. In this case, the patient presented only toothache and maxillofacial swelling at first, then fever and headache appeared 1 week later. With the progress of the disease, the patient presented brainstem involvement such as extending tongue to the left and speaking unclearly and black eschars were also observed around the eyes and the external auditory canal. Soon after, he progressed to brainstem failure. Consistent with literature reports, the onset of typical lesions is late and associated with poor prognosis.

### Survival Situation

The overall survival rate for ROCM is about 85% (5), and the main factors affecting the survival rate include the time of diagnosis, type of diagnosis, treatment methods (9). Yohai pointed out that if the delay between diagnosis and treatment of mucormycosis exceeds 6 days, survival rates will be significantly reduced (5). Anand analyzed 224 cases of mucormycosis reported since 1943, of which 123 (55%) had a 67% reduction in fatality due to early diagnosis and early surgical treatment (10). Therefore, the timing of diagnosis may directly affect patient survival. Delayed treatment, such as the appearance of intracranial involvement, results in a significantly reduced prognosis.

### Diagnostic Method

However, the diagnosis of fungal infection of central nervous system is difficult, which requires comprehensive analysis of history, epidemiology, basic diseases, clinical manifestations, imaging manifestations and laboratory examination results (11, 12). The commonly used clinical diagnostic methods include the following:

**Routine and biochemical examinations of CSF:** It analyzes possible pathogens by measuring the patient's intracranial pressure and the levels of cells, proteins, glucose and chloride in the CSF. However, the specificity of this method is poor, and sometimes false negative results may occur.

**Etiological examination of CSF:** It involves microscopic examination and culture. However, due to the influence of clinical use of antibiotics, the detection rate is low, and the requirements for specimen collection and transportation are strict, and the accuracy of the diagnosis of bacteria with similar phenotypes and biochemical characteristics is poor. For mucormycosis, its growth rate is slow, so the culture cycle is long, which is not conducive to early diagnosis (13–15).

**Blood tests:** It includes blood smear, pathogen culture, serum antigen and antibody detection, etc. Its diagnostic significance is equivalent to that of CSF.

**Immunological examination:** Common methods include galactomannan antigen test (GM test), (1, 3) – β-D – dextran test (G test), antibody test, etc. The principle is to identify the pathogen by measuring the cell wall or antigen of the fungus, but there are some defects, such as high false positive rate, difficult to distinguish the past infection from the current infection. Because the cell wall of mucor contains little or almost no (1, 3) – β-D – glucan, the result of this test was negative in our patient.

**Imaging examination:** Imaging examination is helpful for the detection of mucosal edema and thickening, extraocular muscle and soft tissue swelling, bone destruction, brain parenchymal lesions, intracranial vascular occlusion and other conditions in the affected areas before the appearance of clinical symptoms, and can also be used to judge the therapeutic effect and determine surgical methods. Patient's CT showed paranasal sinusitis and bilateral hypertrophic inferior turbinate and his MRI showed multiple intracranial soft tissue infections, suppurative otitis media, and gingival infection. All of his imaging findings are not typical and have poor clinical suggestive effect.

**Molecular biological examination:** The commonly used clinical molecular diagnostic method is PCR, which is more sensitive than pathogen culture (16–18) and has a shorter detection time (19, 20). However, false negative results may occur when the DNA content is low, and false positive results may occur when the DNA of other fungi is interfered. Due to the lack of uniform standards and clinical validation (21), the application of this technique in patients with mucormycosis is rarely reported (22, 23), and is still under study.

**Histopathological examination:** Pathological results obtained by puncture of intracranial lesions can indicate the type of pathogenic bacteria, and mycelium found by local tissue biopsy is the gold standard for the diagnosis of mycormycosis (24–26). However, this procedure is invasive and is poorly tolerated by patients.

In a study of suspected central nervous system infections in adults, only 25% of cases resulted in a clear etiological diagnosis (27). Failure to obtain clear etiological evidence will affect the implementation of precise antimicrobial therapy, lead to unnecessary use of antibiotics, increase the risk of bacterial resistance, and increase the medical cost. In our case, In the early stage of the disease, there was no obvious suggestive evidence, and the traditional etiological test was negative. However, because the therapeutic effect of broad spectrum antibiotics was not good, we timely took the cerebrospinal fluid and sent it to mNGS for detection, and the result detected *Lichtheimia ramosa*, which confirmed the diagnosis of ROCM in combination with clinical manifestations. In conclusion, mNGS can be used as an effective complementary means for etiological diagnosis when traditional methods cannot be used in time (28).

### Clinical Application of mNGS

In a retrospective study of organ transplant recipients, Palacios identified a new virus using mNGS techniques, which were subsequently validated by pathogen culture, PCR, immunohistochemistry and serological analysis (29). This study is the first to show that mNGS can be used as a new tool to identify novel pathogens.

mNGS is a rapid diagnostic method which has a wide application prospect. Unlike PCR, mNGS does not require primers or specific amplification and is less affected by the use of antibiotics and the body's immune status (30). It can directly sequence the base sequences in the samples (31) and obtain the genome sequences of a variety of pathogens that may be infected in the samples by one-time detection, which is characterized by rapid and high accuracy (32). Therefore, it can be used to cultivate pathogens that are difficult to find or have a relatively low culture rate or a long period of time (33), predict infection in advance by detecting microecology (34), predict drug sensitivity (31), interpret the diversity and abundance of pathogen population, and explore the relationship between pathogen, environment and host. At the same time, mNGS can be considered as an alternative non-invasive testing tool for the detection of infections that cannot tolerate invasive testing.

#### Application Status

Currently, mNGS can detect more than 6,350 bacteria, 1,798 viruses, 10,664 fungi and 234 parasites (30, 35–37). In recent years, researchers have used mNGS technology to provide diagnostic basis for human herpesvirus, pseudorabies virus, cysticercosis, toxoplasma gondii and other infections (38), and it can guide the treatment effectively. In addition, when mNGS detects related pathogens, the CSF culture program can be adjusted to improve the positive rate of related pathogens culture. What's more, reliable negative results also play a role in clinical diagnosis and treatment, helping to reduce the concern of clinicians about active infection.

#### Applications in the Central Nervous System

A growing number of studies in recent years have shown that mNGS play a key role in pathogen recognition of central nervous system infections (28, 39, 40). When the clinical symptoms are atypical, the traditional etiological detection methods may omit some pathogens that have not been considered, but mNGS technology can make a supplement to reduce the rate of missed diagnosis. This technology also plays an important role in the identification of difficult-to-diagnose infections, such as fever of unknown origin, repeated negative clinical tests, or unknown pathogens. A growing number of cases and studies are demonstrating the great potential of mNGs, which are changing the way clinicians think about treating infections caused by rare pathogens, from traditional medicine to genome-based diagnostics.

Li et al. evaluated the diagnostic efficacy of mNGS in central nervous system infections and found that the sensitivity of mNGS was 61.74% and the specificity was nearly 100%, the area under the ROC curve was 0.81 (41), which indicate that mNGS has a strong diagnostic ability in infectious diseases of the central nervous system. Compared with CSF culture, mNGS can indicate central nervous system infection with a higher accuracy and a shorter time (about 36 hours), which provides a new diagnostic clue for patients with immunodeficiency or severe diseases that are difficult to diagnose.

### Diagnosis and Treatment Experience

In this case, the early medication was not targeted and did not successfully prevent the progression of the disease. It was not until the mNGS in the CSF indicated the infection of *Lichtheimia ramosa* that the patient was finally diagnosed. However, due to a variety of factors such as late diagnosis, intracranial lesions indicated by MRI and large necrotic area which makes it difficult for drugs to penetrate, the overall prognosis of patients is poor. It suggests that the following issues should be paid attention to in the process of clinical diagnosis and treatment: (1) The susceptible people should consider this disease when they have symptoms such as fever, headache, swelling of maxillofacial region and nasal congestion and should carry on the etiology examination and the tissue biopsy pathology examination in time to achieve early diagnosis and treatment. (2) The discovery of mucor's characteristic by biopsy is the gold standard for diagnosis of this disease, but medication can be taken as soon as possible according to clinical experience, so as not to delay diagnosis and treatment. (3) For patients with intracranial infection with atypical symptoms and rapid disease progression, the pathogen must be identified as soon as possible, and mNGS has a certain auxiliary role in diagnosis.

In conclusion, specific pathogens should be highly suspected based on the patient's underlying disease history, clinical manifestations, and ineffective use of conventional antibiotics. However, due to the diversity of pathogens, the limitations of traditional detection methods, and the uneven judgment of clinicians on target pathogens, many infectious diseases can not be identified in clinical practice. Traditional etiological detection methods are time-consuming and have low sensitivity, which cannot meet the needs of early diagnosis and treatment of this kind of disease. Sensitive, rapid, accurate and simple diagnostic techniques are needed. In recent years, the emerging mNGS technology has helped to improve the level of diagnosis, and has played a role in the detection of a variety of pathogens. While the technique is currently used only as a second-line option in cases where testing for other pathogens is inconclusive, but with the rapid advances in genomics and sequencing, mNGS will be more and more widely used in the traceability, detection, typing and drug resistance evaluation of infectious diseases, and will develop rapidly in the direction of fast and economic development.

## Data Availability Statement

The datasets presented in this study can be found in online repositories. The names of the repository/repositories and accession number(s) can be found in the article/supplementary material.

## Ethics Statement

The studies involving human participants were reviewed and approved by First Affiliated Hospital of Zhengzhou University. The patients/participants provided their written informed consent to participate in this study. Written informed consent was obtained from the individual(s) for the publication of any potentially identifiable images or data included in this article.

## Author Contributions

YCL, JZ, and YGL analyzed and interpreted patient data. BH, LJD, and ZYS performed the experiments. YCL, CCW, and MX analyzed the genomics data. YCL and BH wrote the manuscript. All authors read and approved the final manuscript.

## Funding

This research was equally funded and supported by Henan Province Science and Technology Project Grant 192102310054. Fund of the First Affiliated Hospital of Zhengzhou University 2018020136.

## Conflict of Interest

The authors declare that the research was conducted in the absence of any commercial or financial relationships that could be construed as a potential conflict of interest.

## Publisher's Note

All claims expressed in this article are solely those of the authors and do not necessarily represent those of their affiliated organizations, or those of the publisher, the editors and the reviewers. Any product that may be evaluated in this article, or claim that may be made by its manufacturer, is not guaranteed or endorsed by the publisher.
